# Influencing factors of sleep quality in pregnant: a structural equation model approach

**DOI:** 10.1186/s40359-024-01657-1

**Published:** 2024-03-25

**Authors:** Mailiman Bahani, Yuxia Zhang, Yufeng Guo, Salawati Haretebieke, Di Wu, Liping Zhang

**Affiliations:** 1 College of Public Health, Xinjiang Medical University, Urumqi, China; 2 Department of Clinical Nutrition, Urumqi Maternal and Child Health Institute, Urumqi, China; 3 College of Medical Engineering and Technology, Xinjiang Medical University, Urumqi, China

**Keywords:** Pregnancy, Sleep quality, Anxiety, Depression, Structural equation model

## Abstract

**Background:**

To describe the changes in the level of sleep quality during pregnancy among pregnant women in Urumqi; also to construct a structural equation model of the factors influencing the sleep quality of pregnant women, to analyze the path relationship between the influencing factors, and to take reasonable interventions to prevent and reduce the occurrence of sleep disorders among pregnant women.

**Methods:**

986 pregnant women who gave birth in Urumqi Maternal and Child Health Hospital and Urumqi Youai Hospital between August 2021 and May 2023 were selected. The Pittsburgh Sleep Quality Index (PSQI), Self-Rating Anxiety Scale (SAS), and Edinburgh Postpartum Depression Scale (EPDS) were used to assess the sleep, anxiety, and depression of pregnant women, Building a structural equation model based on AMOS 23.0 Tools.

**Results:**

The results of structural equation modeling showed that: basic characteristics, obstetrical characteristics, tocolysis, lifestyle, psychological had a direct effect on the PSQI, with path coefficients of 0.243, 0.106, 0.140, 0.174, 0.658, the corresponding T-values for each path are 4.585, 2.677, 2.578, 2.297, and 9.036. The indirect effect of basic characteristics, obstetric characteristics and lifestyle on PSQI was 0.123, 0.020, 0.027.

**Conclusions:**

The high incidence of sleep disorders in pregnant women and their close association with psychological symptoms in pregnant women should focus on screening and counseling regarding psychological disorders in pregnant women, thus improving the quality of sleep in pregnant women.

## Introduction

Sleep is an indispensable life activity for human beings and an important protective mechanism, occupying almost one-third of life. Adequate and good sleep can satisfy both physical and psychological needs of human beings, as well as allow people to recover physical and mental strength and enhance work efficiency. Pregnancy is a short and unique process, due to the hormone levels during pregnancy affecting pregnant women in physical, physiological, social and psychological changes, making pregnant women more prone to insomnia, urinary frequency, frequent waking up at night and sleep deprivation, which can lead to sleep disorders and adverse pregnancy outcome [1]. In addition, socio-demographic characteristics (age, education level, economic income and BMI), mood disorders have been reported to affect the quality of sleep in pregnant women, with higher depression and anxiety scores associated with poorer sleep quality [2, 3].

Most pregnant mothers report altered sleep during pregnancy, and results from previous studies have shown a wide range in the prevalence of poor sleep quality in women during pregnancy, ranging from 29–84% [4, 5],with a mean PSQI score of 6.07 [5.30, 6.85] during pregnancy [4],Sleep quality is characterized by poorer subjective sleep quality, reduced habitual sleep efficiency, and sleep disturbances increase, and longer time to fall asleep. Chronic low-quality sleep exacerbates the risk of multiple negative perinatal pregnancy outcomes, including mood disorders [5, 6], cesarean section rate, prolonged labor and delivery, gestational diabetes mellitus [7], preeclampsia, preterm labor, and low birth weight of neonates [1, 8, 9]. It is thus clear that sleep disorders pose a serious threat to the health of both the pregnant woman and the fetus.

Given the complex interplay of these factors, a comprehensive analysis was deemed necessary to understand the current status and influences on sleep quality in pregnant women. Structural equation modeling (SEM) provides a valuable statistical method to synthesize path-reflective relationships between variables and identify the direct and indirect effects of multiple factors on sleep quality. In this study, a theoretical model was initially constructed using sleep quality as an endogenous latent variable and basic characteristics, obstetrical characteristics, tocolysis, lifestyle, and psychological status as exogenous latent variables, and the fit of the theoretical model was verified to explain and predict the factors affecting sleep quality in pregnant women to ensure that the model proposed by the investigators was supported by data under real conditions. In turn, the key factors leading to poor sleep quality were identified, healthcare professionals can implement targeted interventions and develop strategies to promote better sleep during pregnancy in order to improve the overall health of pregnant women during gestation, optimize their pregnancy experience, while promoting healthy fetal development in the womb.

## Methods

### Participants

The present study was a cross-sectional study in which pregnant women in pregnancy who underwent labor checkups at the obstetrics outpatient clinics of Urumqi Maternal and Child Health Hospital and Urumqi You’ai Hospital during the period of August 2021 ∼ May 2023 were selected by simple random sampling, and pregnant women who met the inclusion criteria were surveyed face-to-face with an electronic questionnaire. Firstly, the purpose, procedure and filling requirements of this survey were explained to pregnant women, and after fully understanding this study, they signed the informed consent form. At the beginning of the survey, pregnant women could choose to fill out the questionnaire independently in the hospital public number with their cell phones, and professionally trained investigators were beside them to explain the entries that were not understood by the research subjects in time; for pregnant women who were not able to answer the questionnaire independently on their cell phones, they could also choose to fill out the paper questionnaire, and fill out the questionnaire in the form of a one-question-one-answer format between the investigators and the pregnant women.

### Inclusion criteria

1. Pregnant women aged ≥ 18 years;
2. Volunteered to participate in this research study and was able to complete the electronic questionnaire independently;
3. Local permanent residents (≥ 1 year of residence).

### Exclusion criteria

1. Suffering from a serious mental illness;
2. Planned early termination of pregnancy;
3. Incomplete information on the questionnaire.

### Data collection tools

#### Basic information questionnaire

The Basic Information Questionnaire collects basic information on pregnant women’s gestation period, age, height, weight, ethnicity, literacy, place of residence, occupation, husband’s information, per capita monthly household income, past medical history, maternal history, exercise, health behaviors, husband-wife relationship, in-laws’ relationship, and whether they are sociable or not.

#### Pittsburgh sleep quality index scale

The Pittsburgh Sleep Quality Index (PSQI) was used to evaluate the sleep of pregnant women [4]. The scale is a questionnaire containing 18 entries to measure habitual sleep quality over a recent month [10]; it consists of seven subscales assessing sleep duration, nocturnal sleep disturbances, sleep latency, sleep quality, daytime dysfunction, sleep medication use and sleep efficiency. Scores for each subscale ranged from 0 to 3, and overall scores ranged from 0 to 21. Higher scores indicate poorer sleep quality in women during pregnancy. In this study PSQI score greater than 7 indicates poor quality of sleep and less than or equal to 7 is good quality of sleep [11].The Cronbach’s α was 0.721.

#### Self-rating anxiety scale

The Self-Rating Anxiety Scale (SAS) was used to assess anxiety symptoms in pregnant women [12]. The scale consists of 20 items, and the scores of each item are summed to obtain a crude score, which is multiplied and rounded to 1.25 to obtain a standardized total score (SAS). In this study, a SAS greater than or equal to 50 was defined as the presence of anxiety symptoms, with higher scores indicating a more pronounced anxiety condition [13]. The Cronbach’s α was 0.733.

#### Edinburgh postnatal depression scale

The Edinburgh Postpartum Depression Scale (EPDS) was applied to assess depression during pregnancy [14], the scale score is obtained by summing the 10 entries included, with a total score ranging from 0 to 30, and a total EPDS score of greater than or equal to 10 was used in the study as the threshold for screening for depressive symptoms [15]. The Cronbach’s α was 0.808.

### Statistical analysis

Survey data were entered using the Epidata 3.1 database, and statistical analyses were performed using IBM SPSS Statistics 27.0 (IBM Corporation, Armonk, NY, USA) and AMOS 23.0 (IBM, New York, NY, USA). For measurement information, expressed as mean ± standard deviation, t-test was used for comparison between two groups and analysis of variance (ANOVA) was used between multiple groups if normal distribution and chi-square were met; rank-sum test was used when normal distribution was not met. For the count data, expressed as frequency and percentage, the $\chi^{2}$ test was used for comparison between groups. to describe the current epidemiologic status of sleep quality among pregnant women in Urumqi and the prevalence trends on different demographic characteristics. Spearman correlation was applied to analyze the correlation between variables. The main influences on sleep quality of pregnant women and the path relationships between them were explored through structural equation modeling, and the following goodness-of-fit indices were used to evaluate the model: λ²/df < 5, CFI > 0.90,GFI > 0.90, AGFI > 0.90, IFI > 0.90, and RMSEA < 0.05.

### Moral statement

Participation in the study was voluntary and anonymous, and participants were given the option to opt out at any time while undergoing the survey to ensure that personal privacy was respected and protected. Prior to the start of the survey, all subjects signed a written informed consent to participate in the program. The study involving human subjects in this protocol was reviewed and approved by the Nutrition and Health Ethics Committee of the Chinese Center for Disease Control and Prevention (No. 2021-008), in accordance with the Declaration of Helsinki.

## Results

### Basic characteristic

Table 1 describes the basic characteristics of the included study population, which included 986 pregnant women, of whom 217, 471, and 298 were in the early, middle, and late stages of pregnancy, respectively. The age ranged from 20 to 47 years, with a mean age of 31.28 ± 4.25 years, 620 (62.9%) were Han, and 312 (31.6%) were predominantly educated to undergraduate level. There were 39.0% of pregnant women who had their first pregnancy and 232 (23.5%) who were on birth control. The PSQI score of pregnant women was 4.84 ± 3.12, 179 (18.2%) pregnant women had sleep disorders, 111 (11.3%) and 297 (30.1%) had symptoms of anxiety and depression, 48 (43.2%) pregnant women had both anxiety and sleep disorders, 104 (18.2%) pregnant women had both depression and sleep disorders, and there was a significant difference between both and the non-sleep disordered group. disorder group were significantly different.

**Table 1:** Comparison of basic characteristics of pregnant women with and without sleep disorders

Varibale	Group	n (%)	PSQI ≤ 7	PSQI > 7	λ²/F	*P*
Age(years)		31.28 ± 4.253			1.098	0.334
pregnancy	1st	217 (22.0)	185 (85.3)	32 (14.7)	7.515	0.023
	2st	471 (47.8)	393 (83.4)	78 (16.6)		
	3st	298 (30.2)	229 (76.8)	69 (23.2)		
BMI (kg/m2)	≥ 24	556 (56.4)	450 (80.9)	106 (19.1)	0.711	0.399
	< 24	430 (43.6)	357 (83.0)	73 (17.0)		
Ethnic	Others	366 (37.1)	307 (83.9)	59 (16.1)	1.621	0.203
	HAN	620 (62.9)	500 (80.6)	120 (19.4)		
Age(years)	≥ 35	145 (14.7)	110 (75.9)	35 (24.1)	4.097	0.043
	< 35	841 (85.3)	697 (82.9)	144 (17.1)		
Education level	Primary school	38 (3.9)	32 (84.2)	6 (15.8)	18.673	0.002
	middle college	165 (16.7)	151 (91.5)	14 (8.5)		
	senior high school	174 (17.6)	147 (84.5)	27 (15.5)		
	junior college	256(26)	207 (80.9)	49 (19.1)		
	Undergraduate course	312 (31.6)	240 (76.9)	72 (23.1)		
	postgraduate	41 (4.20)	30 (73.2)	11 (26.8)		
Occupation	unemployed	221 (22.4)	188 (85.1)	33 (14.9)	1.990	0.158
	employed	765 (77.6)	619 (80.9)	146 (19.1)		
Monthly income /(yuan)	< 1,000	12(1.1)	10 (83.3)	2 (16.7)	6.667	0.353
	1,000–1,999	24 (2.4)	20 (83.3)	4 (16.7)		
	2,000–2,999	191 (19.4)	160 (83.8)	31 (16.2)		
	3,000–4,999	311 (31.5)	246 (79.1)	65 (20.9)		
	5,000–9,999	340 (34.5)	286 (84.1)	54 (15.9)		
	> 10,000	91 (9.2)	74 (81.3)	17 (18.7)		
	unclear	17 (1.7)	11 (64.7)	6 (35.3)		
Primiparity	No	385 (39.0)	330 (85.7)	55 (14.3)	6.362	0.012
	Yes	601 (61.0)	477 (79.4)	124 (20.6)		
Gravidity	> 1	517 (52.4)	438 (84.7)	79 (15.3)	6.041	0.014
	≤ 1	469 (47.6)	369 (78.7)	100 (21.3)		
Parity	≥ 1	407 (41.3)	348 (85.5)	59 (14.5)	6.241	0.012
	< 1	579 (58.7)	459 (79.3)	120 (20.7)		
Vaginal Bleeding	No	830 (84.2)	689 (83.0)	141 (17.0)	4.802	0.028
	Yes	156 (15.8)	118 (75.6)	38 (24.2)		
Tocolytic	No	754 (76.5)	633 (84.0)	121 (16.0)	9.569	0.002
	Yes	232 (23.5)	174 (75.0)	58 (25.0)		
Smoke	No	961 (97.5)	787 (81.9)	174 (18.1)	0.059	0.808
	Yes	25 (2.5)	20 (80.0)	5 (20.0)		
Exercise	No	792 (80.3)	641 (80.9)	151 (19.1)	2.251	0.134
	Yes	194 (19.7)	166 (85.6)	28 (14.1)		
Alcohol	No	981 (99.5)	803 (81.9)	178 (18.1)	0.012	0.915
	Yes	5(0.5)	4 (80.0)	1 (20.0)		
Husband drinking alcohol	No	706 (71.6)	589 (83.4)	117 (16.6)	4.187	0.041
	Yes	280 (28.4)	218 (77.9)	62 (22.1)		
Anxiety	No	875 (88.7)	744 (85.0)	131 (15.0)	52.989	0.001
	Yes	111 (11.3)	63 (56.8)	48 (43.2)		
Depressed	No	689 (69.9)	614 (89.1)	75 (10.9)	81.338	0.001
	Yes	297 (30.1)	193 (81.8)	104 (18.2)		

Table 2 shows the overall score, component scores and depression and anxiety scores of sleep quality in women in early, mid and late pregnancy. As the course of pregnancy increased, both sleep quality scores and depression and anxiety scores showed an increasing trend, with significant differences in overall sleep quality scores across trimesters of 4.22 (3.13), 4.81 (2.97), and 5.34 (3.25), and depression scores of: 6.25 (4.90), 7.56 (4.56), and 7.56 (4.92). There were significant differences in all the indicators except Anxiety, Sleep latency, Use Hypnotic drug, Daytime dysfunction.

**Table 2:** Comparison of sleep quality, depression, and anxiety rate scores by gestational week

Variable	Total	1st^a^	2st^b^	3st^c^	F	*P*
PSQI	4.84 (3.12)	4.22 (3.13)	4.81 (2.97)	5.34 (3.25)	8.247	< 0.001
EPDS	7.27 (4.77)	6.25 (4.90)	7.56 (4.56)	7.56 (4.92)	6.413	0.002
SAS	40.37 (8.18)	39.60 (8.23)	40.39 (8.29)	40.91 (7.95)	1.612	0.200
subjective sleep quality	0.91 (0.72)	0.76 (0.73)	0.92 (0.70)	1.00 (0.73)	7.402	0.001
sleep latency	0.91 (0.84)	0.85 (0.87)	0.90 (0.82)	0.97 (0.84)	1.372	0.254
sleep duration	0.35 (0.70)	0.25 (0.60)	0.32 (0.68)	0.47 (0.78)	6.619	0.001
sleep efciency	0.38 (0.74)	0.27 (0.59)	0.38 (0.76)	0.46 (0.80)	4.274	0.014
sleep disturbance	1.13 (0.64)	0.96 (0.64)	1.15 (0.61)	1.23 (0.64)	11.884	< 0.001
use Hypnotic drug	0.02 (0.20)	0.01 (0.10)	0.02 (0.21)	0.03 (0.23)	0.547	0.579
daytime dysfunction	1.14 (1.01)	1.12 (1.10)	1.12 (0.98)	1.18 (1.00)	0.421	0.657

Note:a:First trimester; b:Second Trimester;c:Late pregnancy

### Correlation analysis

Table 3 shows Spearman’s correlation between socio-demographic factors, depression and anxiety and sleep quality among all participating pregnant women (*n* = 986). Sleep quality was correlated with education level (*r* = 0.129,*p* < 0.001), parity (*r* = 0.080,*p* < 0.05), gravidity, primiparity, vaginal bleeding, tocolysis (*r* = 0.099,*p* < 0.001), husband drinking alcohol, anxiety (*r* = 0.232,*p* < 0.001), depressed (*r* = 0.287,*p* < 0.001) were correlated. The higher the level of education, the greater the correlation between the emergence of sleep quality problems in pregnant women. Similarly, the more pronounced the expression of depression and anxiety, the increase in sleep disorders in women during pregnancy is associated. The strength of the correlation between these variables was between weak and moderate, and there was no correlation between the quality of sleep of pregnant women and their occupation and monthly income.

**Table 3:** Correlation analysis between basic characteristics of pregnant women

variable	Education level	0ccupa-tion	Monthly income	Primiparity	Gravidity	Parity	Vaginal Bleeding	Tocolytic	Smoke	Husband drinking alcohol	Alcohol	PSQI	Anxiety
Occupation	0.264**												
Monthly income	0.358**	0.188**											
Primiparity	0.328**	0.068*	0.137**										
Gravidity	0.275**	0.103**	0.096**	0.642**									
Parity	0.312**	0.063*	0.217**	0.773**	0.535**								
Vaginal Bleeding	0.054	-0.074*	0.032	0.096**	-0.001	0.070*							
Tocolytic	0.136**	-0.017	0.054	0.121**	-0.035	0.164**	0.434**						
Smoke	0.028	0.025	0.112**	-0.030	-0.024	0.109**	-0.017	0.093**					
Husband drinking alcohol	0.126**	0.053	0.114**	0.048	0.013	0.112**	0.041	0.075*	0.127**				
Alcohol	0.045	0.004	-0.025	0.013	0.075*	0.002	0.008	0.028	0.079*	0.113**			
PSQI	0.129**	0.045	0.006	0.080*	0.078*	0.080*	0.070*	0.099**	0.008	0.065*	0.003		
Anxiety	0.017	0.030	-0.071*	0.002	0.027	-0.066*	-0.005	-0.008	-0.057	-0.004	0.020	0.232**	
Depressed	0.086**	0.051	0.026	0.027	0.030	0.088**	0.055	0.037	0.049	0.091**	-0.016	0.287**	0.347**

Notes:**: *P* < 0.001; *: *P* < 0.05

### Structural equation model

**Fig. 1:**
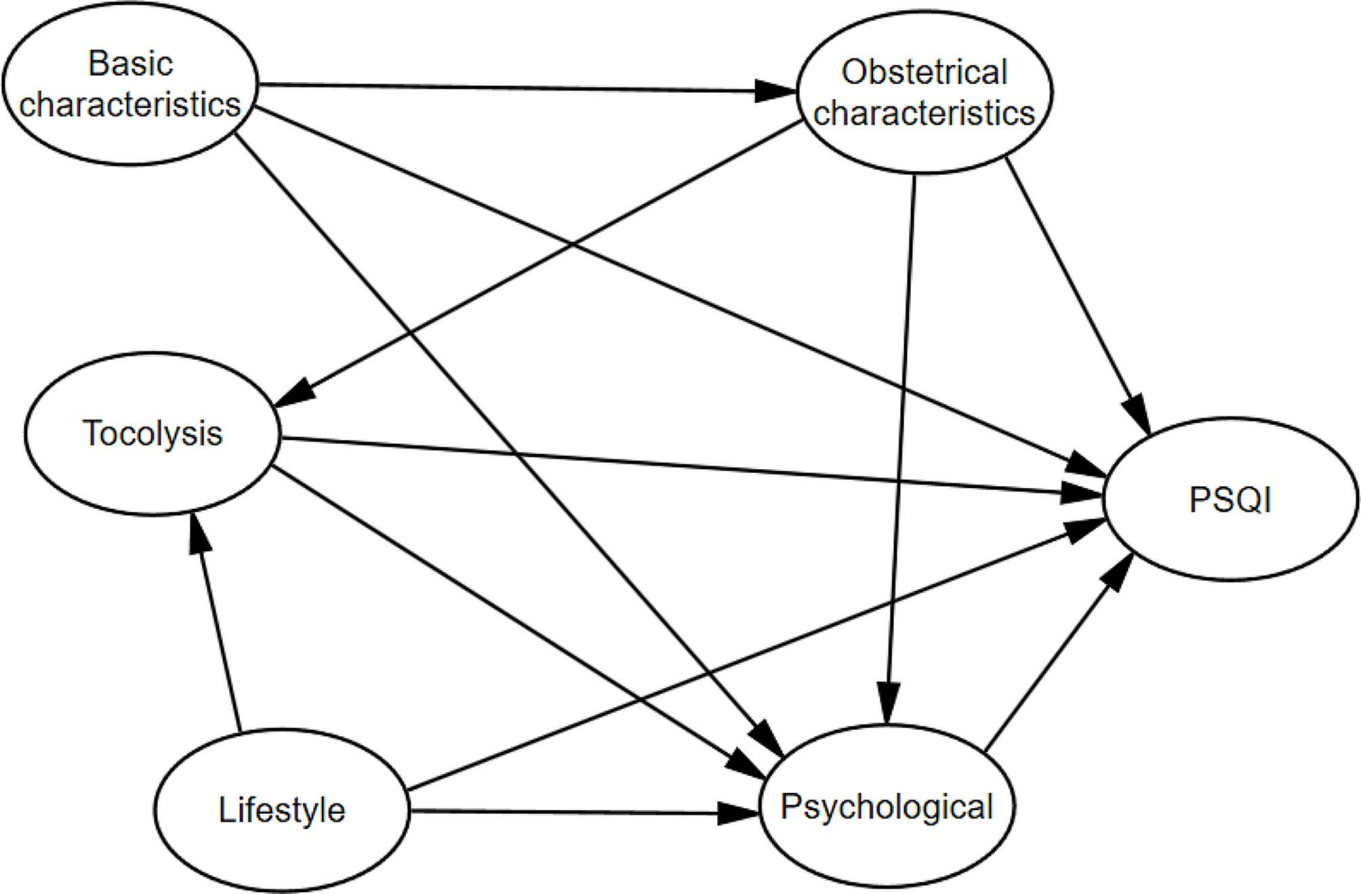
Theoretical model of sleep quality in women during pregnancy

In order to reasonably and comprehensively study the changes in the level of sleep quality during pregnancy among pregnant women in Urumqi; and to explore the influencing factors of sleep quality among women during pregnancy, an initial structural equation model was established based on the results of previous studies in the relevant literature. Inclusion of study variables in exploratory factorial molecules. KMO test and Bartlet’s Sphericity test, the KMO value was 0.649 and the Bartlet ‘s sphericity test had a difference (P < 0.001) and the results showed that the data satisfied the factor analysis. The principal component analysis method was used to extract factors, in order to make the difference between the attributes of the factors is obvious, using the maximum variance rotation method to rotate the factors, the cumulative explanation of 61.62% of the variance, to get the rotated factor loadings as in Table 4, factor 1 is named obstetric characteristics (Primiparity, Parity, Gravidity), factor 2 is named basic characteristics (Monthly income, Occupation, Educatin level), factor 3 is named as tocolysis (Vaginal Bleeding, Tocolytic), factor 4 is named as psychological (Anxious, Depressed), factor 5 is named as Lifestyle (Smoke, Alcohol, Husband drinking alcohol). The initial structural equation modeling of the five variables of basic characteristics, obstetric characteristics, tocolysis, lifestyle, and psychological with sleep quality was established. As shown in Fig. 1.

**Table 4:** The rotated component matrix

Variables	Factor 1	Factor 2	Factor 3	Factor 4	Factor 5
Primiparity	0.916				
Parity	0.838				
Gravidity	0.831				
Monthly income		0.720			
Occupation		0.664			
Educatin level		0.636			
Vaginal Bleeding			0.831		
Tocolytic			0.828		
Anxious				0.829	
Depressed				0.793	
Smoke					0.643
Alcohol					0.628
Husband drinking alcohol					0.618

Extraction method: Principal Component Analysis

Rotation method: Caesar normalization maximum variance method

The rotation has converged after 5 iterations

**Fig. 2:**
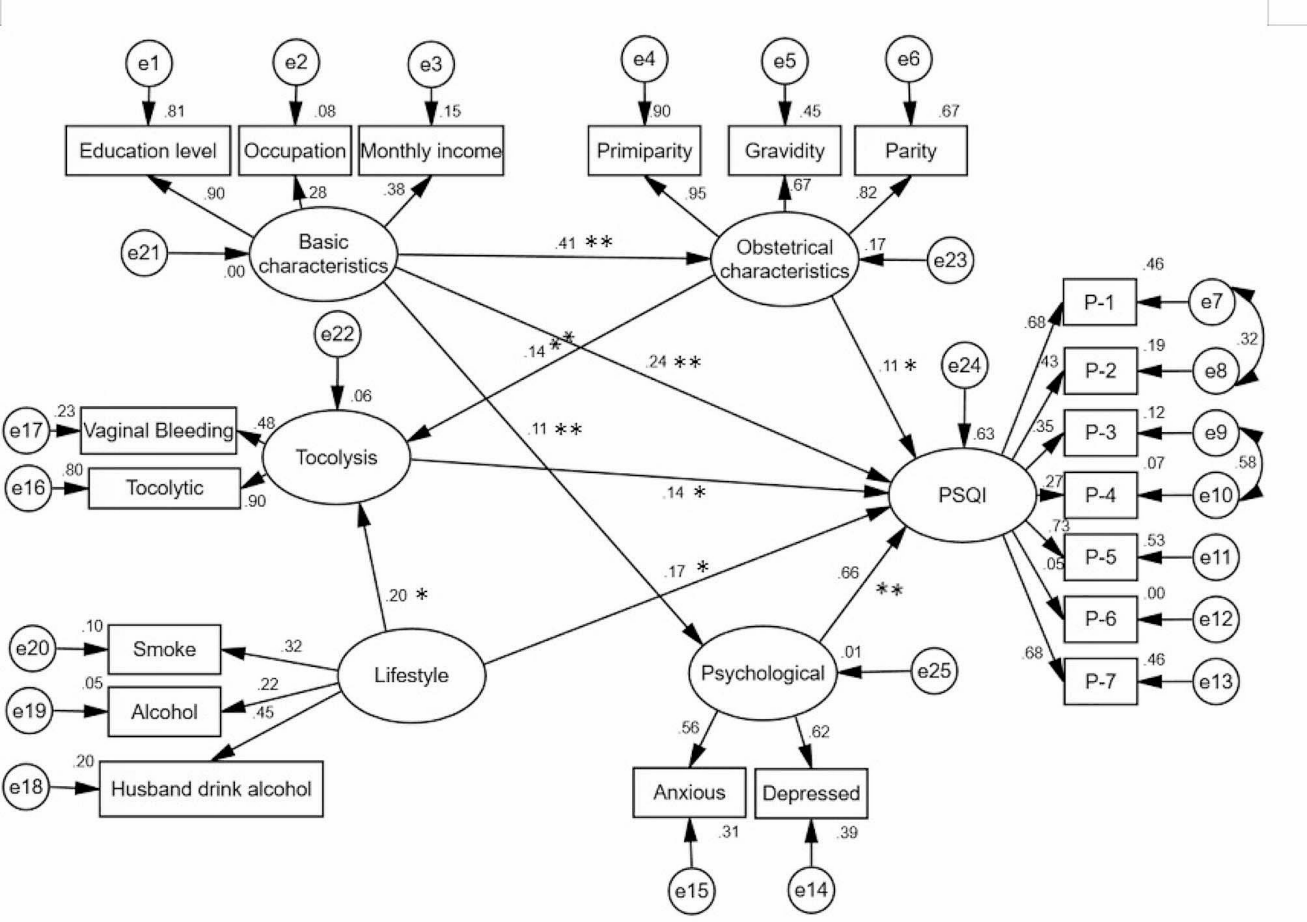
Structure equation model of sleep quality in pregnant women AMOS pathway diagram Note: P-1: Subjective sleep quality, P-2: Sleep latency, P-3:Sleep duration,P-4:Sleep duration, P-5: Sleep disturbance, P-6: Use Hypnotic drug,P-7: Daytime dysfunction Notes:**P < 0.001;* *P* < 0.05.

The initial model is tested and corrected, and the maximum likelihood method is chosen for parameter estimation. Comprehensive consideration of the correction index, deletion of meaningless paths and other measures after repeated modifications, the SEM model with a better fit is shown in Fig. 2. The results of the main fitting indexes show that: Chi/df = 3.038,GFI = 0.951,AGFI = 0.935,IFI = 0.926,TLI = 0.910,RMSEA = 0.046.The specific results are shown in Table 5.

**Table 5:** Model fitness results for structural equation modeling

Fit Index		Chi/df		GFI		CFI		AGFI			TLI		IFI		RMSEA	
Reference value		1 to 5		> 0.9		> 0.9		> 0.9			> 0.9		> 0.9		< 0.05	
Initial value		5.816		0.914		0.824		0.882			0.778		0.825		0.070	
Correction value		3.038		0.951		0.925		0.935			0.910		0.926		0.046	

Note: GFI:goodness of fit index,CFI: comparative fit index, AGFI: adjusted goodness of fit index,TLI: Tucker-Lewis index,IFI: incremental fit index,RMSEA:.root mean square error of approximation

The summary table of path coefficients shows that there is a direct effect of obstetric characteristics → PSQI with a path coefficient of 0.106 and a t-value of 2.677 (*P* < 0.001), a positive and direct effect of obstetric characteristics → tocolysis with a path coefficient of 0.142 and a t-value of 3.876 (*P* < 0.001), and a direct and positive effect of tocolysis → PSQI with a path coefficient of 0.140 and a t-value of 2.578 (*P* < 0.05). The standardized path coefficient of basic characteristics on PSQI through tocolysis was 0.020 (0.14*0.14), the indirect effect was 0.020, the direct effect was 0.106, and the total effect was 0.126. The direct and indirect effects accounted for 84.1% and 15.9% of the total effect, respectively. Thus, 15.9% of the effect of obstetrical characteristics on PSQI was through tocolysis. The direct effect of basic characteristics → PSQI was 0.243 with a t-value of 4.548 (*P* < 0.001) and the indirect effect was 0.123; psychological → PSQI had a direct effect with a path coefficient of 0.658 and a t-value of 9.036. lifestyle → PSQI had a path coefficient of 0.174 and a t-value of 2.297 (*P* < 0.05). The indirect effect of lifestyle on pregnant women’s sleep quality through birth control was 0.027,accounting for 13.43% of the total effect. See Table 6.

**Table 6:** Structural equation modeling path coefficients

Model paths			S.E.	C.R.	*P*	Direct effect	Indirect effect	Total effect
Obstetrical_characteristics	←	Basic_characteristics	0.022	7.412	**	0.410	—	0.410
Psychological	←	Basic_characteristics	0.126	2.138	*	0.108	—	0.108
Tocolysis	←	Obstetrical_characteristics	0.030	3.876	**	0.142	—	0.142
Tocolysis	←	Lifestyle	0.181	2.04	*	0.196	—	0.196
PSQI	←	Tocolysis	0.070	2.578	*	0.140	—	0.196
PSQI	←	Basic_characteristics	0.023	4.548	**	0.243	0.123	0.366
PSQI	←	Obstetrical_characteristics	0.042	2.677	*	0.106	0.02	0.126
PSQI	←	Psychological	0.125	9.036	**	0.658	—	0.658
PSQI	←	Lifestyle	0.183	2.297	*	0.174	0.027	0.201

Notes:**P < 0.001;* *P* < 0.05

## Discussion

Sleep disorders are common throughout pregnancy. Previous national and international studies have shown that pregnant women have varying degrees of sleep quality problems during all trimesters [16]. In this study, the mean PSQI score of all subjects was 4.84 ± 3.12, and 18.2% of pregnant women had sleep disorders. It was lower than the findings of Li [17]. who had a sleep disorder rate of 54.3%, and this difference may come from the difference in the threshold value of PSQI (PSQI > 5). Where the scores were 4.22 ± 3.13, 4.81 ± 2.97, and 5.34 ± 3.25 in early, middle, and late pregnancy, respectively, we found that PSQI scores increased with the progression of pregnancy, and that pregnant women’s sleep quality was most susceptible to disturbances in late pregnancy. Similar to the findings of Kızılırmak’s study [18]. This increasing trend may be related to poor sleep quality due to hormone levels, fetal movement, physical discomfort, and frequent nighttime bathroom visits during pregnancy [19, 20].

In this study, we found that the quality of sleep in pregnant women was also associated with age, education, whether they were primiparous, pregnancy history, and birth control. The prevalence of sleep disorders in pregnant women of higher gestational age (age ≥ 35) was 24.1%, which was higher than that of lower age, which was 17.1%. That is, the higher the maternal age, the higher the prevalence of sleep disorders during pregnancy [16, 21]. It may also be related to the fact that older women are themselves more susceptible to workplace pressures, caring for their families and children, and the concern about the higher risk of adverse pregnancy outcomes associated with advanced age, which makes their psychological burden heavier and affects their sleep [22]. We found that there is also a close relationship between the level of education of pregnant women and sleep status, undergraduate and postgraduate there is a sleep retardation rate of 49.9%, high educational level but affect the quality of sleep, to consider that it may be related to the probability of this group of people to engage in mental labor is relatively large, the pressure of the work is high, so they are more susceptible to the impactt. There is a discrepancy with the findings of Cai[5], who concluded that the higher the level of education, the more knowledge and access pregnant women acquire to regulate their sleep, leading to better sleep improvement.

The present study further explored the direct and indirect effects of sleep quality in pregnant women based on structural equation modeling with the aim of providing better interventions for better improvement of sleep quality during pregnancy. The results of the study showed that basic characteristics, obstetric characteristics,tocolysis, lifestyle, psychological had a direct effect on PSQI. tocolysis had a mediating effect between obstetric characteristics and PSQI, indicating that obstetric characteristics not only directly affect PSQI, but also indirectly affect sleep quality through tocolysis. The mediating effect was 15.9%. Parity and pregnancy history were risk factors for sleep quality. Compared to primiparous women, transient women reported poorer sleep quality, which may be related to the physical and psychological stress that exists during pregnancy itself, the fact that women also have to take care of their first child and the expectation of the gender of the second child in some families, which makes the pregnant women anxious, thus leading to poorer quality of sleep [23–25]. Van [26]compared the effect of comparing the presence or absence of a history of miscarriage on the quality of sleep in the later part of pregnancy in pregnant women who were pregnant during the late stage of pregnancy. showed that sleep duration (7.1 ± 1.1 h) and sleep efficiency (83.7 ± 7.9%) were similar in both groups, however, the PSQI score of the miscarriage group (7.8 ± 2.6) was significantly lower than that of the control group (6.7 ± 3.1), suggesting that having a history of miscarriage is an influencing factor in the low quality of sleep in women with a late pregnancy. At the same time, when pregnant women with vaginal bleeding and their own low back and abdominal pain receive tocolysis treatment, anxiety, anxiety and other adverse psychological emotions due to the fear of their own and fetal health, leading to poor treatment compliance and affecting the effect of sleep quality. Therefore, it is necessary to pay attention to the pregnancy history of pregnant women, and adopt the treatment of Tocolytic for pregnant women with threatened abortion, along with certain psychological guidance, to prevent the occurrence of adverse pregnancy outcomes [27].

Lifestyle has a direct effect on sleep quality (0.174), can also indirectly affect the quality of pregnant women’s sleep from tocolysis(0.027), the total effect is( 0.201). Alcohol not only harms the drinker, in the case of alcohol consumption, alcohol fluctuates moods domestic violence will rise, resulting in harm to family members and the atmosphere, foreign scholars have shown that if the husband is drinking, the woman is more likely to be subjected to domestic violence, more susceptible to physical and psychological stress, which further affects sleep [28–30].

In addition, pregnancy sleep quality during pregnancy is strongly associated with pregncancy’s psychological, and we found that psychological had a direct and significant effect on the quality of sleep of pregnant women with a path coefficient of 0.658. Based on previous studies, it was found that pregnant women experience high mood swings throughout pregnancy, which are accompanied by varying degrees of anxiety and stress as well as depression, and the higher the level of depression in pregnant women, the worse the quality of sleep [31, 32]. Polo-Kantola [33] confirmed a correlation between sleep quality and depression and anxiety during pregnancy. LI found a direct effect of anxiety on depression, suggesting that pregnant women with symptoms of anxiety during pregnancy have a higher risk of developing depressive symptoms [34], and therefore anxiety is one of the strongest factors that have been strongly associated with prenatal depression one of the strongest associations with prenatal depression [35]. Another study shows that depression and anxiety scores in pregnant women are important hubs for regulating the relationship between objective and subjective sleep [36]. Many pregnant women after pregnancy will have concerns about their competence as a mother, worry about their inability to complete the role transition, worry about their delivery and infant feeding methods and the hopes of relatives for pregnant women can produce pregnancy-related anxiety and anxiety, which [37], if appropriate interventions are not taken, will potentially lead to adverse consequences of postpartum depression.

## Conclusions

The results of this study indicate that sleep quality problems are prevalent among pregnant women in Urumqi and increase with the trimester of pregnancy. Considering the path relationships between the variables, basic characteristics, obstetric characteristics, tocolysis, lifestyle、psychological were found to be influential factors for sleep quality, especially the impact of psychology on the sleep quality of pregnant women is particularly significant. Therefore, healthcare organizations should pay attention to screening pregnant women for sleep disorders and psychological disorders, and develop reasonable and effective interventions to reduce the incidence of adverse pregnancy outcomes caused by sleep disorders.

### Limitations

Our research also has certain limitations. Firstly, due to cross-sectional design, it is limited to comprehensively explore the influencing factors of sleep quality in pregnant women; Secondly, our research did not consider social support factors, which may have a certain impact on our results; Finally, the scale for evaluating sleep quality, depression and anxiety in this study was objectively filled out by pregnant women, and there may be some recall bias.

## Data Availability

The authors will supply the relevant data in response to reasonable requests.
